# Supplementary material for: Characterization of Leptoglossus occidentalis Eggs and Egg Glue
Source: Insects. 2023 Apr 19;14(4):396. doi: 10.3390/insects14040396 (PMC10143709; doi:10.3390/insects14040396)
Supplement: Supplementary file 1 [file insects-14-00396-s001.zip › insects-2324995-supplementary.pdf]

SUPPORTING INFORMATION

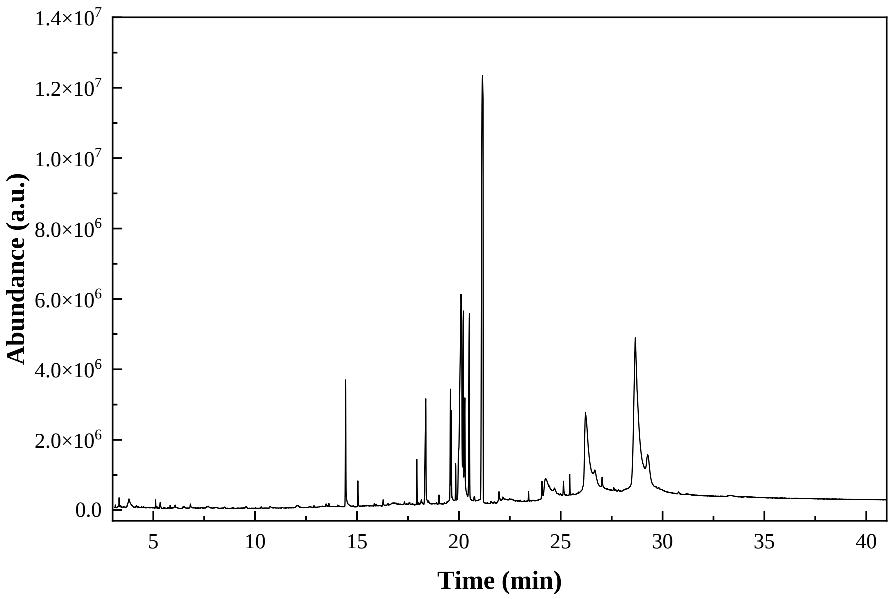

Figure S1. GC−MS chromatogram of *L. occidentalis* eggs.

Table S1. Main chemical species identified in *L. occidentalis* eggs by GC−MS.

Retention time (min)	Area (%)	Assignment	Qual
14.4471	3.7856	Dibutyl itaconate	94
17.9371	1.0225	Hexadecanoic acid, methyl ester	99
18.3763	4.9311	n-Hexadecanoic acid	99
19.5871	3.0710	9,12-Octadecadienoic acid, methyl ester	99
19.6346	2.7232	9-Octadecenoic acid, methyl ester	99
19.8483	1.1173	Methyl stearate	99
20.1094	22.5155	9,12-Octadecadienoic acid	99
20.2222	5.9806	1-Propene-1,2,3-tricarboxylic acid, tributyl ester	95
20.2934	4.9070	Octadecanoic acid	99
20.513	7.1539	Butyl citrate	91
21.1599	33.2528	Tributyl acetylcitrate	87
21.979	1.4978	9-Octadecenamide	94
22.4954	0.3430	Heptaethylene glycol monododecyl ether	58
25.1366	0.7980	13-Docosenamide	94
25.4453	0.6382	Squalene	99

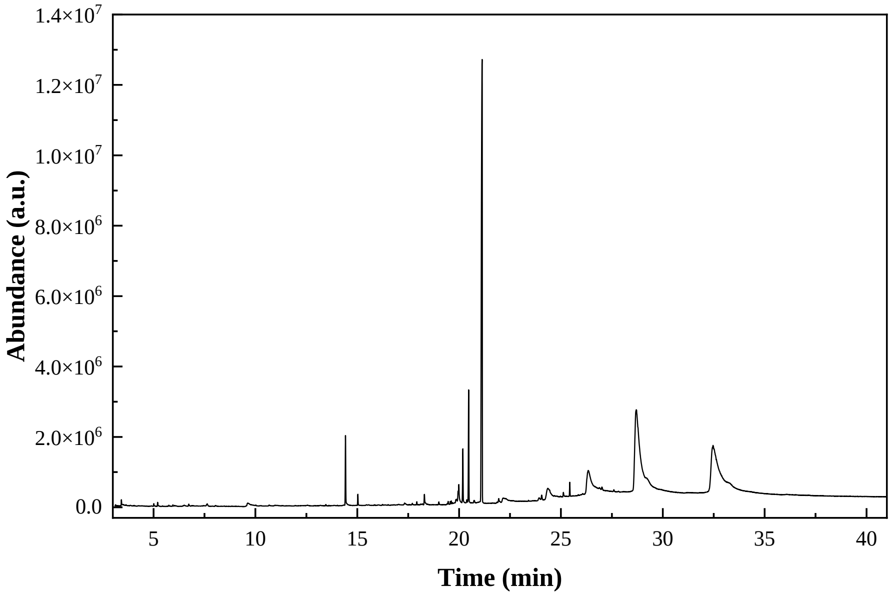

Figure S2. GC–MS chromatogram of *L. occidentalis* glue.

Table S2. Main chemical species identified in *L. occidentalis* egg glue by GC-MS.

Retention time (min)	Area (%)	Assignment	Qual
5.2058	0.3082	1,2-Cyclopentanedione	90
5.9537	0.0918	Hexanoic acid	47
9.6395	1.5604	Dianhydromannitol	97
14.4293	4.7458	Dibutyl itaconate	94
16.2455	0.1078	Tetradecanoic acid	94
17.9311	0.2009	Hexadecanoic acid, methyl ester	99
18.1092	0.0870	Ethyl 9-hexadecenoate	41
18.2991	1.1017	n-Hexadecanoic acid	99
19.5693	0.1872	9,12-Octadecadienoic acid-, methyl ester	99
19.6167	0.2230	9-Octadecenoic acid, methyl ester	98
19.8719	1.0999	Adipic acid, 2-ethylhexyl isobutyl ester	64
19.9907	3.1248	Oleic Acid	99
20.1865	3.8039	1-Propene-1,2,3-tricarboxylic acid, tributyl ester	95
20.3824	0.3458	Hexadecanamide	55
20.4714	7.3203	Butyl citrate	91
20.7504	0.3095	Tributyl acetylcitrate	30
21.1302	54.1289	Butyl citrate	72
21.9493	1.8635	9-Octadecenamide	93
24.3650	6.5063	Heptaethylene glycol	81
25.1188	0.8805	13-Docosenamide	64
25.4393	1.1882	Squalene	99
